# Migration intensity has no effect on peak HIV prevalence: an ecological study

**DOI:** 10.1186/1471-2334-14-350

**Published:** 2014-06-24

**Authors:** Chris Kenyon, Robert Colebunders, Helene Voeten, Mark Lurie

**Affiliations:** 1 Sexually Transmitted Infections, HIV/STI Unit, Institute of Tropical Medicine, Antwerp, Belgium; 2 Infectious Diseases, University of Antwerp (UA), Head clinical HIV/STI Unit, Institute of Tropical Medicine, Antwerp, Belgium; 3 Research Infectious Disease Control, Municipal Public Health Service Rotterdam-Rijnmond, Rotterdam, the Netherlands; 4 Department of Public Health, Erasmus MC, University Medical Center Rotterdam, Rotterdam, the Netherlands; 5 Epidemiology, Brown University School of Public Health, Providence, RI, USA; 6 Department of Medicine, University of Cape Town, Cape Town, South Africa

**Keywords:** Ecological, Individual-level, Circular migration, International migration, Internal migration, HIV

## Abstract

**Background:**

Correctly identifying the determinants of generalized HIV epidemics is crucial to bringing down ongoing high HIV incidence in these countries. High rates of migration are believed to be an important determinant of HIV prevalence. This study has two aims. Firstly, it evaluates the ecological association between levels of internal and international migration and national peak HIV prevalence using thirteen variables from a variety of sources to capture various aspects of internal and international migration intensity. Secondly, it examines the relationship between circular migration and HIV at an individual and population-level in South Africa.

**Methods:**

Linear regression was used to analyze the association between the various measures of migration intensity and peak national HIV prevalence for 141 countries and HIV prevalence by province and ethnic group in South Africa.

**Results:**

No evidence of a positive ecological association between national migration intensity and HIV prevalence was found. This remained the case when the analyses were limited to the countries of sub-Saharan Africa. On the whole, countries with generalized HIV epidemics had lower rates of internal and external migration. Likewise, no association was found between migration and HIV positivity at an individual or group-level in South Africa.

**Conclusion:**

These results do not support the thesis that migration measured at the country level plays a significant role in determining peak HIV prevalence.

## Background

Population mobility is commonly identified as a key driver of the HIV epidemic [1]. Decosas et al. for example argue: “the fact that population movements distribute HIV is secondary to the fact that certain types of migration cause HIV epidemics” [2]. What is the evidence to back up this assertion? There are numerous plausible pathways whereby migrants can be put at risk of HIV [3]. One is that individual migrants are separated from their partners and social support networks and therefore more likely to adopt additional sex partners to those at home [4]. Numerous, but not all [3,5,6], individual-level studies have shown a higher prevalence of HIV in migrants and/or their partners compared to non-migrants [7-10]. This evidence does not however establish that migration is a significant determinant of population level HIV prevalence.

The reason for this is related to the increasingly appreciated fact that the differential spread of HIV around the world can be better understood by considering both individual-level and population-level perspectives. As an example, individual risk factors such as lifetime number of sex partners have been shown to be a determinant of who within a population is likely to contract HIV [11]. However, people living in the 20 countries with generalized HIV epidemics (GHEs - defined as countries with an HIV prevalence ≥ 5% in 15–49 year olds) [12] do not have a higher number of lifetime partners compared to those in the rest of the world [13]. Instead population level factors such as those which increase the connectivity of the sexual networks, may be important drivers of HIV spread in GHEs [14]. Because these population level factors are properties of populations not reducible to those of individuals, it is necessary and appropriate to evaluate them at the ecological level [3,14,15].

A number of papers have made the “atomistic fallacy” [16] of inferring that migration is a driver of HIV prevalence based on individual-level studies showing a correlation between migration and HIV prevalence [17-23]. This inference fallacy has then led some authors to conclude that migration is central to the genesis of GHEs. Some researchers, for example, deduce from an individual-level study showing an association between migration and HIV incidence that “young women engaging in sex with migrant men is the key driver in the spread of HIV infection (in Southern Africa)” [23].

A key step to showing that migration is a determinant of population HIV prevalence would be to demonstrate that the relevant form of migration was more common in populations with higher HIV prevalence rates. There has however only been one published study that has evaluated the relationship between migration intensity and HIV prevalence at a population level [24]. In this study one of the current authors (Voeten) found evidence of a strong association between *recent urban migration of women* (percentage of 15–49 year old women who migrated into major cities in 28 African countries in the preceding 12 months) and urban antenatal HIV prevalence in the same year (or if this was not available then adjacent years). Since the publication of this study, a good case has been made for the use of peak HIV prevalence rather than HIV prevalence at-the-date-the-exposure-variable-is-measured as the outcome variables in ecological studies of the determinants of differential HIV spread [12,25,26]. In short, a significant advantage of peak HIV prevalence is that it avoids the HIV-introduction-time bias. The year that HIV prevalence peaked in countries with GHEs varied considerably and in large part this was related to the different times that HIV was introduced into particular populations [27]. Given that persons with HIV live for around ten years even in the absence of treatment, a country’s HIV prevalence at a particular point represents the product of the interactions of the various component causes over the previous decade or longer [27]. If the exposure variable was measured early in the HIV epidemic and an ecological study uses date-of-exposure variable ascertainment as the basis for determining HIV prevalence then this HIV prevalence may be misleadingly low. This is exemplified in the previous migration study where a number of countries were represented with relatively low HIV prevalences because the migration variables were measured early in their epidemics [24]. Namibia for example was apportioned an HIV prevalence of 4.2%, which is what HIV prevalence was in 1992 when the migration measurements were taken, rather than its peak of 16.5%. Peak HIV prevalence avoids this HIV introduction time bias and by its nature is a summary measure of the interactions of the composite causes of HIV spread over the past decade and longer. As long as the prevalence of the exposure variable is relatively stable over time then it will be more informative to relate this exposure variable to peak HIV prevalence rather than date-of-exposure variable HIV prevalence.

This study has two aims. Firstly, it evaluates the strength of the association between levels of internal and international migration and peak HIV prevalence in 141 countries using five publicly available databases. Secondly, it examines the relationship between circular migration and HIV prevalence within South Africa. A number of HIV epidemiologists have postulated that circular migration is the most important form of migration that promotes the spread of HIV [4,10]. Because comparable cross-country estimates of circular migration do not exist [28], in the second part of the study, we examine the relationship between circular migration and HIV in both South Africa’s provinces and ethnic groups. Adult HIV prevalence varies by up to a factor of five between South Africa’s nine provinces and a factor of 40 between its ethnic groups [29]. High migration intensity is one of the factors that have been put forward to explain differential HIV spread in the country [23]. We assessed the association at both an individual- and population-level between this kind of migration and HIV prevalence in South Africa.

## Methods

### Cross-country comparisons

The first part of the study is an ecological analysis (at the country-level) comparing various indicators of migration intensity with peak HIV prevalence.

### Peak HIV prevalence

The Joint United Nations Programme on HIV/AIDS (UNAIDS) provided country-specific age-standardized HIV seroprevalence per 100 adults 15–49 years old for the years 1990-2009 [30]. These estimates are based on the best available evidence from population-based testing, epidemic modeling and older antenatal clinic surveillance estimates. From these data we derived the peak HIV prevalence for each of the 149 countries listed in this report between the years 1990 and 2009.

### Internal migration

The United Nations Development Programme (UNDP) periodically estimates country-specific data on the *internal migration rate*[28]. This was defined as the percentage of persons who had moved during the course of their lives within the borders of their country (usually measured across regional, district or municipal boundaries) resulting in a change of usual place of residence. The data were from 51 countries taken from censuses and household surveys between 1990 and 2005.

A more comparable but less complete set of indicators was obtained from the World Bank’s World Development Report, 2009 [31]. The authors obtained internal migration-related indicators from nationally representative household surveys from 35 developing countries on whom they could find good comparable data. The surveys were done between 1992 and 2006. Internal migrants were defined as individuals who are not living in the same district in which they were born. This definition did not count returnees as migrants – that is persons who moved away from their place of birth in the past, but returned by the time of the survey.

We used two indicators from this source. The *percent internal migrants*, was defined as the percentage of internal migrants in the country’s working-age population.

The *percent recent internal migrants* was defined as the percentage of the total working-age population made up of internal migrants who had moved into the new area within five years before the year of the survey.

Bell is one of a number of leading theorists in migrancy studies who have argued cogently that comparisons of migration intensity based on league tables comparing the proportions of people moving within selected countries, as reported in censuses and surveys are suboptimal for two main reasons [32]. Firstly, there are often significant differences in the time period when migration is measured between the various countries. Secondly, differences in statistical geography between countries can create problems for comparisons. This relates to the fact that the number of migrants recorded in any form of data collection is dependent on the number and shape of the units into which a territory is divided. This can be illustrated by comparisons of migration intensities for countries x and y who have the same size and numbers of inhabitants, but x is divided into 1000 zones and y into 10 zones. This zonation system will make it more likely that routine measures of migration will describe migration rates as higher in x than y even if there is no difference in the rate of movement or distance moved by the populations in the two countries. To circumvent these problems of comparability Bell proposes using certain conventional measures of migration intensity and Courgeau’s k index- a statistic which adjusts migration intensity for differences in statistical geography as described below [32].

The *Crude Migration Intensity* (CMI), is defined as the total number of internal migrants (M) in a given time period as a percentage of the population at risk (P) such that CMI = 100 M/P.

*Courgeau’s Index k* was originally used in 1973 as a means of comparing migration among countries with different territorial divisions. It is computed as: CMI = k log n2, where n represents the number of regions in the zonal system, and k is the slope of a regression line for various n and CMI, that reflects the overall intensity of migration at various spatial scales. It has been shown to provide an excellent synthetic index of migration intensity [33]. We use the values computed by Bell et al., of Courgeau’s k index and the CMI from a study of 27 countries (5 developed and 22 developing) that had recently completed their national censuses in 2009 [33].

The data were taken from Demographic and Health Surveys from the respective countries. We repeated the same linear regression analysis using the same datasets but using peak HIV prevalence as the outcome variable and using in-migration prevalence closest to the year of peak HIV as the exposure variable.

### International migration

The UNDP provided three indictors of international migration [28]. The *immigration rate*, was defined as the percentage of a country’s total population that was made up of international migrants. Data for only two time-points were provided, 1960 and 2005, both of which we used. The *emigration rate* was defined as the percentage of the population in the country that had emigrated as of 2000–2002. The *international movement rate* refers to the sum of total stock of immigrants into and emigrants from a particular country as of 2000 to 2002. It is defined as the percentage of the sum of a country’s resident population and its emigrant population in this period. This indicator was calculated primarily based on data from censuses conducted between 1995 and 2004. In cases where census data were not available, the authors of the UNDP used data from population registers or other sources were used [28].

The *average annual net migration rate* was provided by the United Nations, Department of Economic and Social Affairs. This is defined as the annual number of immigrants minus emigrants for the period 2005–2010 (per 1000 inhabitants) [34].

With the exception of the two measures computed by Bell (the CMI and Courgeau’s Index k), each of the migration indicators was taken from a published report from a multinational agency – either a United Nations- or a World Bank-based institution. The migration variables used, the sources they were taken from and the years the data were collected from are detailed in Table 1.

**Table 1:** Sources of the migration variables and year the data was collected

**Variable**	**Source of variable**	**Years data collected**
**Internal migration**		
Internal migration rate	Human Development Report, 2009 [28]	1990-2005
Percent internal migrants	World Development Report, 2009 [31]	1992-2006
Percent recent internal migrants	World Development Report, 2009 [31]	1992-2006
Recent urban migration (Women)	Voeten et al. [24]	1987-2005
Recent urban migration (Men)	Voeten et al. [24]	1987-2005
Crude migration intensity (5 year)	Bell et al. [33]	2008-2009
Crude migration intensity (Lifetime)	Bell et al. [33]	2008-2009
Courgeau’s k index (5 year)	Bell et al. [33]	2008-2009
Courgeau’s k index (Lifetime)	Bell et al. [33]	2008-2009
**International migration**		
Average annual net migration rate	United Nations Department of Economic and Social Affairs: World Population Prospects [34]	2000-2005
Immigration rate (1960)	Human Development Report, 2009 [28]	1960
Immigration rate (2005)	Human Development Report, 2009 [28]	2005
Emigration rate	Human Development Report, 2009 [28]	2000-2002
International movement rate	Human Development Report, 2009 [28]	2000-2002
**Within South Africa**		
Spent ≥ 1 month living in different province	National HIV Prevalence, HIV Incidence, Behaviour and Communication Survey I [35]	2002
Born in different province	National HIV Prevalence, HIV Incidence, Behaviour and Communication Survey I [35]	2002
Spent ≥ 1 month away from home in the preceding year	National HIV Prevalence, HIV Incidence, Behaviour and Communication Survey II [36]	2005

### Circumcision prevalence

A number of other factors have been proposed to explain the differences in HIV prevalence between countries. These include the prevalence rates of circumcision [37], other STIs and especially HSV-2 [38], condom use [36] and the effectiveness of STI treatment [39]. Of these the only indicator with high quality data and global coverage is the national prevalence rates of male circumcision. In our multivariate analysis we therefore only control for circumcision prevalence. The prevalence rates of circumcision, as of December 2006, were taken from a publication from the World Health Organization and Joint United Nations Programme on HIV/AIDS which estimated national circumcision prevalence rates [40]. Countries were classified as having circumcision prevalence rates <20%, 20-80% or >80%. These estimates were based on Demographic and Health Survey data where available, or otherwise from other published sources. In the case of four countries no data was available and these countries were dropped from the multivariate analyses.

### Migration and HIV within South Africa

We assessed the association at both an individual- and population-level between circular migration and HIV prevalence in two South African data sets. These were the National HIV Prevalence, HIV Incidence, Behaviour and Communication Survey’s I and II (SABSSM I & II) conducted in 2002 and 2005. Both surveys used a multi-stage stratified sampling approach. When correctly weighted to account for the complex sampling design and HIV testing non-response, the samples were representative of the population of South Africa for the main reporting domains of sex, age, race and province. Structured questionnaires were used to collect demographic, social and behavioral data. Migrants were defined in SABSSM II as those respondents who said they spent at least one month away from home in the preceding year. SABSSM I asked respondents somewhat different questions and migrants were defined in two ways - those who were born in a different province to that they were currently living or those who had spent a month or more living in a different province at some stage in their lives. Each respondent’s ethnicity was defined based on their answers to two questions, “What is your race?” and “What is the main language you speak at home?” Individuals were classified as black, white, coloured and Indian based on the first question. The black group was then subdivided according to the main language spoken at home. Because SABSSM I & II were not designed to be representative for small black ethnic groups, we have limited our analysis to black ethnic groups that comprise more than five percent of the total population of South Africa. These are the Isixhosa, Isizulu, Sesotho, Setswana and Sepedi groups.

The SABSSM II sample consisted of 23,275 individuals in 10,584 households. 10,584 out of 12,581 (84.1%) of households agreed to participate in the study and in each of these households a maximum of three individuals was eligible to participate. 23,276 of the 24,236 (96%) individuals who were eligible from these households participated in the interview. 15,851 agreed to be tested for HIV (73.3% of those aged over 15 years old). We limited our analysis to the 13,884 individuals aged 15 to 55 years old who agreed to HIV testing. For more detailed information about the survey methodology see Shisana et al. [35,36].

### Ethics statement

The data used in this study were analyzed anonymously, using publicly available secondary data, therefore no ethics approval was deemed necessary for this work.

### Statistical analyses

For all the indicators of international and internal migration we related peak HIV prevalence to migration using linear regression. Categorical variables were compared using the chi-square test. All of our analyses of SABSSM I and II are weighted with sampling weights correcting for sample design and appropriate wave non-response. All analyses were performed using STATA 12 (Stata, East College Station, TX).

## Results

### Cross-country comparisons

**Figure 1:**
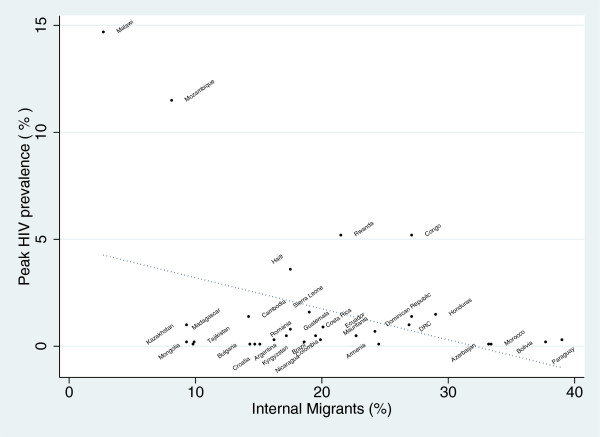
**Association between national peak HIV prevalence and percent of the working age population that have migrated to a different district to that they were born in.** (R^2^ - 0.149; *P* = 0.038. Migration data from nationally representative household surveys from 35 developing countries [31]. Peak HIV prevalence data from UNAIDS [30]).

As shown in Table 2, two out of nine indicators of internal migration intensity and two out of four indicators of international migration were significantly associated with peak HIV prevalence. In all cases the association was negative and of a weak magnitude. Controlling for the effect of circumcision prevalence made little difference. As shown in Figure 1, the weak negative associations were determined largely by the large leverage effect of a number of countries with GHEs but low migration rates.

**Table 2:** Uni- and multivariate (controlling for prevalence of circumcision) regression analyses of the relationship between the migration variables and country-level peak HIV prevalence

			***Univariate***			***Multivariate***	
**Variable**	***N***^**a**^	**No. (total) GHEs > median**^**b**^	**Coefficient**	**R**^**2**^	***P-value***	***Coefficient***	***P-value***
**Internal migration**							
**Internal migration rate**	51	1 (7)	-0.166	0.093	0.028	-0.167	0.027
**Percent internal migrants**	29	2 (4)	-0.145	0.149	0.038	-0.150	0.033
**Percent recent internal migrants**	28	2 (4)	-0.133	0.038	0.316	-0.143	0.289
**Recent urban migration (Women)**	28	9 (16)	0.470	0.084	0.132	0.396	0.198
**Recent urban migration (Men)**	22	7 (12)	0.314	0.050	0.316	0.331	0.157
**Crude migration intensity (5 year)**^**d**^	11	0 (0)	0.010	0.002	0.878	0.001	0.980
**Crude migration intensity (Lifetime)**	20	1 (4)	-0.124	0.025	0.503	-.089	0.594
**Courgeau’s k index (5 year)**	16	1 (1)	4.13	0.149	0.140	4.27	0.115
**Courgeau’s k index (Lifetime)**	15	0 (2)	-0.91	0.065	0.359	-0.533	0.582
**International migration**							
**Average annual net migration rate**	93	2 (13)^c^	0.12	0.000	0.808	0.052	0.901
**Immigration rate (1960)**	126	13 (19)	-0.004	0.000	0.950	-0.003	0.966
**Immigration rate (2005)**	141	5 (19)	-0.072	0.017	0.113	-0.071	0.125
**Emigration rate**	141	3 (19)	-0.162	0.042	0.014	-0.165	0.015
**International movement rate**	141	2 (19)	-0.109	0.046	0.010	-0.108	0.012

^a^The column heading **“***N***”** refers to the number of countries included in linear regression analyses. The observations were limited for most variables. See methodology section for definitions of the variables.

^b^The column “No. (total) GHEs > median” refers to the number of countries with generalized HIV epidemics (GHEs) where the dependent variable is higher than the median for the dependent variable of the countries analyzed (total No. of countries with GHEs represented in the analysis).

^c^Because this variable reflects the net migration rate, it ranges from -38 to +37. 2. Two out of the 13 countries with GHEs had migration rates below the .25 quantile or above the .75 quantile and this result is reported here.

^d^Crude migration intensity is measured at the provincial or major regional level. The results for the municipal level were similar but are not shown.

The majority of countries with GHEs had migration rates less than the median of all the countries measured (see Tables 2 and 3). This was the case for all the migration variables except for three: immigration rate in 1960, recent urban migration in women and recent urban migration in men.

**Table 3:** Prevalence of peak HIV, circumcision and various indicators of migration intensity by country (see methodology section for definitions of the variables and sources of the data)

**Country**	**Peak HIV (%)**	**Average annual net migration rate (no./1000)**	**Immigration rate -1960 (%)**	**Immigration rate – 2005 (%)**	**International movement rate (%)**	**Internal migration rate (%)**	**Percent internal migrants (%)**	**Crude migration intensity (5 year)**	**Crude migration intensity (Lifetime)**	**Emigration rate (%)**	**Circumcision prevalence (%)**	**Courgeau’s k index (5 year)**	**Courgeau’s k index (Lifetime)**
**Algeria**	0.1	-0.373	4	0.7	6.9					6.2	>80		
**Angola**	2	3	2.4	0.3	5.8					5.5	>80		
**Argentina**	0.5	0	12.6	3.9	5.6	19.9	17.2		19.9	1.6	<20	0.574	
**Armenia**	0.1	-30		16.1	28.1	24.5	24.5			20.3	<20		
**Australia**	0.1	4	16.5	21.3	22.5			10.39		2.2	20-80	1.239	
**Austria**	0.3	6	11.5	14	17.2					5.5	<20		
**Azerbaijan**	0.1	-3		3	15.8	33.2	33.2			14.3	>80		
**Bahamas**	3.9	0	10.3	9.7	19.3					10.8	<20		
**Bangladesh**	0.1	-2	1.2	0.7	5.1					4.5	>80		
**Barbados**	1.4	0	4.2	10.4	36.6	31.1				29.8	<20		
**Belarus**	0.3	0		11.3	26.1	10.8			10.78	15.2	<20		5.751
**Belgium**	0.2	2	4.8	8.5	14.6					4.4	<20		
**Belize**	2.4	4	8.2	14.4	27.4	14.2				16.5	<20		
**Benin**	1.4	-1	1.5	2.4	8.8					7.5	>80		
**Bhutan**	0.2	-38	4.3	5.7	3.8					2.2	<20		
**Bolivia**	0.2	-3	1.3	1.2	5.3	15.2	37.7			4.3	<20		
**Botswana**	26.3	2	1.4	4.4	3.8					0.9	<20		
**Brazil**	0.5	0	1.9	0.4	0.8	10.1	19.5	3.4	10.07	0.5	<20		
**Bulgaria**	0.1	-8	0.3	1.3	11.6	14.3	14.3			10.5	<20		
**Burkina Faso**	4	-3	1.3	5.6	17.9					9.8	>80		
**Burundi**	5.9	-9	4.3	1.1	6.5					5.4	<20		
**Cambodia**	1.4	2	7	2.2	3.9	11.7	14.2		11.65	2.3	<20		1.553
**Cameroon**	5.5	0	3.2	1.2	1.9					1	>80		
**Canada**	0.2	0	15.4	19.5	21.5					4	20-80	0.987	
**CAR**	10.1	1	2.9	1.8	4.2					2.7	20-80		
**Chad**	3.5	-1	1.9	3.6	3.7					3.2	>80		
**Chile**	0.4	1	1.4	1.4	4.5	21.3		9.59	21.27	3.3	<20	1.455	4.159
**China**	0.1	0	0	0	0.5	6.2			6.193	0.5	<20	0.432	
**Colombia**	0.9		0.4	0.3	4.1	20.3	20.1	6.42	20.25	3.9	<20	0.53	2.625
**Comoros**	0.1	-1	0.8	2.2	10.7					7.7			
**Congo**	5.2	1	2.6	3.8	20	27.1	27.1			14.7	>80		
**Costa Rica**	0.3	4	2.5	10.2	9.7	20	19.9	10.6	20.02	2.6	<20	1.268	4.121
**Croatia**	0.1	7		14.9	23.8	26.6	14.7			12			
**Cuba**	0.1	-2	2	0.1	9.6	15.2				8.9	<20		
**Czech Republic**	0.1	0	0.4	4.4	7.7					3.5	<20		
**Côte d'Ivoire**	7.5	1									>80		
**DRC**	1.4						27.1				>80		
**Denmark**	0.2	3	2.1	7.8	10.7					4.3	<20		
**Djibouti**	2.9	9	13.9	13.7	5.8					2.2	>80		
**Dominican Republic**	1	-3	4.3	4.1	10.4	17.7	26.9			9.1	<20		
**Ecuador**	0.5	-1	0.5	0.9	5.9	20.2	22.7	8.25	20.23	5.3	<20	0.864	3.153
**Egypt**	0.1	-4	0.8	0.3	3.1					2.9	>80		
**El Salvador**	0.8	-9	1.2	0.6	14.6	16.7				14.3	<20		
**Equatorial Guinea**	5	6	7.7	1	14.7					14.5	>80		
**Eritrea**	1.2	-1	0.5	0.3	12.8					12.5	>80		
**Estonia**	1.2	0		15	28.5					12.2	<20		
**Ethiopia**			1.7	0.7	1.4					0.4	>80		
**Fiji**	0.1	-10	5.1	2.1	16.6					15	<20		
**Finland**	0.1	2	0.7	3.3	9					6.6	<20		
**France**	0.4	0	7.7	10.6	13.1					2.9	<20		
**Gabon**	5.4	1	4.3	17.9	22.8					4.3	>80		
**Gambia**	2	-2	9.9	15.2	16.4					3.6	>80		
**Georgia**	0.1	-21		4.3	22.1					18.3	<20		
**Germany**	0.1	8	2.8	12.9	15.3					4.7	<20		
**Ghana**	2.3	-1	7.8	7.6	7.3	17.8		5.96	17.75	4.5	>80	0.66	3.127
**Greece**	0.1	9	0.6	8.8	17.2					7.8	<20		
**Guatemala**	0.8	-8	1	0.4	5.2	11.1	17.5			4.9	<20		
**Guinea**	1.8	29	0.4	4.4	14.3					6.3	>80		
**Guinea-Bissau**	2.5	-1	2	1.3	9.9					8.6	>80		
**Guyana**	2.8	-13	2.5	1.3	33.6					33.5	<20		
**Haiti**	3.6	-4	0.4	0.3	8	17.5	17.5			7.7	<20		
**Honduras**	1.5	-5	3	0.4	5.9	17.2	29			5.3	<20		
**Hungary**	0.1	2	5.2	3.3	6.6					3.9	<20		
**Iceland**	0.3		1.9	7.6	16.4					10.6	<20		
**India**	0.4		2.1	0.5	1.4	4.1			4.141	0.8	<20		0.587
**Indonesia**	0.2		2	0.1	1	4.1		2.19	4.07	0.9	>80	0.338	1.318
**Iran**	0.2		0.2	2.9	4.7					1.3	>80		
**Ireland**	0.2		2.6	14.8	28.1					20	<20		
**Israel**	0.2	19	56.1	39.8	40.3					13.1	>80		
**Italy**	0.3	1	0.9	5.2	8.1					5.4	<20		
**Jamaica**	2.2		1.3	1	27					26.7	<20		
**Japan**	0.1	1	0.7	1.6	1.7					0.7	<20		
**Kazakhstan**	1			19.6	35.8	9.3	9.3			19.4	20-80		
**Kenya**	10.5	0	0.7	2.2	2.3	12.6			12.64	1.4	>80		2.524
**Kyrgyzstan**	0.3			5.5	20.6	16.2	16.2			10.5	>80		
**Lao**	0.2		0.9	0.3	6.2					5.9	<20		
**Latvia**	0.7			16.6	33					9.1	<20		
**Lebanon**	0.1	14	8	17.7	27.1					12.9	>80		
**Lesotho**	24.5		0.4	0.3	2.8					2.6	20-80		
**Liberia**	3.6	37	2.7	2.9	7.8					2.7	>80		
**Lithuania**	0.1			4.8	13.9					8.6	<20		
**Luxembourg**	0.3	10	14.8	33.7	38.3					9.5	<20		
**Madagascar**	0.2		2.5	0.2	1.3	9.3	9.3			0.9	>80		
**Malawi**	14.7		8.4	2	3.4	2.7	2.7			1.2	<20		
**Malaysia**	0.5	3	0.7	7.9	10.1	20.7		7.99	20.71	3.1	>80	0.828	
**Maldives**	0.1		1.7	1.1	1.5					0.4			
**Mali**	1.9		3.3	1.4	12.9					12.5	>80		
**Malta**	0.1	3	0.5	2.9	24					22.3	<20		
**Mauritania**	0.7		1.4	2.2	6.3	24.2	24.2			4.1	>80		
**Mauritius**	1	0	1.6	3.3	13.1					12.5			
**Mexico**	0.4		0.6	0.6	9.5	18.5				9	<20	1.364	
**Mongolia**	0.1		0.4	0.4	0.6	9.7	9.8			0.3	<20		
**Morocco**	0.1		3.4	0.2	8.5	33.4	33.4			8.1	>80		
**Mozambique**	11.5	0	0.1	1.9	6	8.1	8.1			4.2	20-80		
**Myanmar**	0.8		1.4	0.2	0.9					0.7	<20		
**Namibia**	16.5		4.5	6.6	8.7					1.3	<20		
**Nepal**	0.5		3.5	3	6.2					3.9	<20		
**Netherlands**	0.2	3	3.9	10.6	14.2					4.7	<20		
**New Zealand**	0.1	7	14.1	20.9	27.3					11.8	<20		
**Nicaragua**	0.2		0.7	0.6	9.6	13.3	18.6			9.1	<20		
**Niger**	1	0	1.7	1.4	5					4	>80		
**Nigeria**	4	0	0.2	0.7	1.4					0.8	>80		
**Norway**	0.1	2	1.7	8	11					3.9	<20		
**Oman**	0.1	6	7.7	25.5	28					0.7	>80		
**Pakistan**	0.1		13	2.1	4.8					2.2	>80		
**Panama**	1.6	1	6.1	3.2	8.2	20.6			20.56	5.7	<20		4.071
**Papua New Guinea**	0.9	0	1	0.4	1.3					0.9	<20		
**Paraguay**	0.3		2.6	2.8	9.8	26.4	39			6.9	<20		
**Peru**	0.5		0.7	0.1	2.9	22.4				2.7	<20		
**Philippines**	0.1		0.8	0.4	5.6	11.7		3.28	11.72	4	20-80	0.337	1.827
**Poland**	0.1		8.2	2.2	7.1					5.1	<20		
**Portugal**	0.6	3	0.4	7.2	21.4	12.8		3.23	12.8	16.1	<20	0.757	3.107
**Qatar**	0.1		32	80.5	60.7					2.3	>80		
**Republic of Korea**	0.1										>80		
**Moldova**	0.4										<20		
**Romania**	0.1		1.8	0.6	5	15.1	15.1			4.6	<20		
**Russian Federation**	1	2	1.4	8.4	15.3					7.7	<20		
**Rwanda**	5.2		1	4.8	3.7	10.4	21.5		10.41	2.7	<20		
**Senegal**	0.9		5.5	2	7					4.4	>80		
**Serbia**	0.1	9	0.9	6.8	18.7					13.6	20-80		
**Sierra Leone**	1.6		2	3	3	19	19			2	>80		
**Singapore**	0.1	14	31.8	35	19.1					6.3	>80		
**Slovakia**	0.1			2.3	10.3					8.2	<20		
**Slovenia**	0.1	4		8.4	7.6					5.2	<20		
**Somalia**	0.7		0.4	0.3	6.7					6.5	>80		
**South Africa**	18.1	3	5.3	2.6	3.9	15.4			15.36	1.7	20-80	1.503	1.817
**Spain**	0.5		0.7	10.7	8.3	22.4			22.37	3.2	<20		3.493
**Sri Lanka**	0.1		10	1.9	6.6					4.7	<20		
**Sudan**	1.1	1	2.1	1.7	3.8					1.7	>80		
**Suriname**	1.1	0	7.7	6.8	36.9					36	<20		
**Swaziland**	25.9		4.9	3.4	4.8					1.1	<20		
**Sweden**	0.1	3	4	12.3	15					3.3	<20		
**Switzerland**	0.4	7	13.4	22.3	26					5.6	<20		
**Tajikistan**	0.2			4.7	16.1	9.9	9.9			11.4	>80		
**Thailand**	2.1		1.8	1.5	2					1.3	<20		
**Togo**	3.6	0	6.5	3.1	6.8					3.7	>80		
**Trinidad and Tobago**	1.5		9.6	2.9	22.8					20.2	<20		
**Tunisia**	0.1		4	0.4	6.3					5.9	>80		
**Turkey**	0.1		3.4	1.9	6					4.2	>80		
**Uganda**	10.7	1	11.4	2.3	2.7	5.2			5.24	0.7	20-80		
**Ukraine**	1.1			11.5	23.8					10.9	<20		
**United Kingdom**	0.2	1	3.2	9.7	14.3					6.6	<20		
**Tanzania**	7.9										20-80		
**USA**	0.6	3	5.8	13	12.4	17.8		6.57	17.84	0.8	20-80	1.267	4.5
**Uruguay**	0.5		7.6	2.5	9.5	24.1				7	<20		
**Uzbekistan**	0.1			4.8	13.4					8.5	>80		
**Viet Nam**	0.4		0	0.1	2.4	21.9		2.9		2.4	<20	0.403	
**Zambia**	15	0	11.9	2.4	5.6					2.2	<20		
**Zimbabwe**	26.5		10.3	3.1	7.4					2.3	<20		

### Migration and HIV in South Africa

At an individual-level, the analysis of SABSSM II found that HIV prevalence was not significantly higher in those who had spent a month or more away from home in the past year (15.5%) compared to those who had not (13.8%; *P* = 0.332). The same was the case when the analysis was restricted to Africans (all black ethnic groups) only. Similarly, the analysis of SABSSM I found that HIV prevalence was not higher in those born in another province (12.8% versus 16.8%; *P* = 0.189) or in those who lived in another province for a period of longer than one month at any stage in the past (14.1% versus 13.4%; *P* = 0.285).

As shown in Table 4, at the inter-ethnic-group-level there was no correlation between HIV prevalence and percentage of the population who had spent at least a month away from home in the past year (R^2^ = 0.18; *P* = 0.284).

**Table 4:** **The prevalence (95% CI) and univariate regression analyses of the relationship of HIV and migration in eight major ethnic groups in South Africa in 2005 based on the SABSSM II**^**a**^

	**No.**	**Age median** **(95% CI)**	**HIV prevalence** **(95% CI)**	**Migration prevalence**^**a**^
**Isixhosa**	1980	29.7 (29.2-30.2)	16.4 (13.4-19.9)	8.1 (6.5-10.2)
**Isizulu**	2071	30.2 (29.8-30.8)	24.4 (21.4-27.8)	10.6 (8.4-13.3)
**Sesotho**	915	31.1 (30.3-31.80	23.8 (18.7-29.3)	11.6 (8.9-14.9)
**Sepedi**	891	29.7 (28.9-30.4)	13.4 (10.6-17.4)	15.0 (11.5-19.3)
**Setswana**	1023	34.6 (33.9-35.3)	15.0 (11.2-19.6)	13.9 (11.5-16.6)
**White**	1402	34.6 (33.9-35.3)	0.5 (0.2-1.0)	10.3 (8.0-13.1)
**Coloured**	2633	31.3 (30.9-31.8)	3.0 (2.1-4.2)	8.2 (6.6-10.2)
**Indian**	1465	32.7 (32.1-33.4)	1.0 (0.3-2.4)	6.7 (5.1-8.8)
**Beta-coefficient**^**b**^			-	1.452
**R**^**2b**^			-	0.18
***P***^**b**^			-	0.284

^a^Migration prevalence defined as the percentage of the ethnic group that spent a period of one month or more living in a different province to their current province in the previous 12 months.

^b^The Beta coefficient, R^2^ and *P* rows represent the univariate regression analyses of the relationship between migration prevalence and HIV prevalence by ethnic group.

## Discussion

When examining 141 countries globally, we found no evidence of a positive ecological association between migration intensity and HIV prevalence. This remained the case when we limited the analyses to 28 countries in sub-Saharan Africa. Of note, our analysis of the relationship between migration and HIV prevalence in sub-Saharan Africa used exactly the same dataset as the study noted above which found a positive association between female in-migration and HIV prevalence [24]. The only difference was in the choice of outcome variable. We used national peak HIV prevalence (derived from UNAIDS estimates of 15–49 years old prevalence) and the original study used HIV prevalence at the time of the survey measuring migration (determined from antenatal surveillance sites). For the reasons outlined above and more fully elsewhere [12], we believe that peak HIV prevalence is a more valid indicator to assess this relationship.

On the whole, countries with GHEs had lower rates of internal and external migration. These results do therefore not support the thesis that migration plays a significant role in the genesis of GHEs at the ecological level.

There are a number of significant limitations that need to be borne in mind when interpreting the cross-country analyses. Firstly, since this part of the study is explicitly ecological, its results pertain to the population and not the individual level. Secondly, a key problem in this type of analysis is the validity of the country-level migration variables. A number of problems have been raised with comparative studies of migration intensity [33]. We have tried to deal with the problems presented by statistical geography by using Courgeaus’s k index. Although this exercise was limited by the low number of countries with available data, adjusting for statistical geography made little difference to the results. It is critical in this kind of assessment that migration intensity is captured at the correct time in relation to peak HIV. This should be during the period of rapid increase in HIV prevalence, and preferably include at least one time-point early during the epidemic take-off. This is a difficult undertaking given the limitations of the available datasets. We attempted to deal with this issue by using variables that are drawn from a long period that includes as much of the 1980–2000 period - when most countries experienced their most rapid increases in HIV prevalence [27]. In addition, the measures of migration utilized in the recent-urban-migration and HIV correlation were all taken from the Demographic and Health Survey at the time that most recently preceded the year of peak HIV prevalence for that country. A third problem is that the data used for the internal-migration rate were obtained by the UNDP from different censuses and surveys using somewhat different questions and at different times and so are not strictly comparable. Fourthly, it is plausible that a particular type of migration is key to the spread of HIV and this is not adequately assessed by the country-level variables. The effect of circular migration was only examined within the South African context. Migration of individuals as opposed to families could plausibly be more important in STI spread. We were unable to assess this. We were also not able to assess any association between HIV prevalence and the frequency of migration. Fifthly, there may be a bias due to an unconsidered confounder. A sixth limitation pertaining to the outcome measure is that not all countries may have reached their peak HIV prevalence. As argued elsewhere, however, the influence of this is likely to be small [12]. Finally, it is possible that migration may exert its effect predominantly, or only, early on in the genesis of an HIV epidemic. If this were the case then our analysis would not be able to isolate this effect.

Our analysis of the relationship between markers of circular migration and HIV within South Africa found no association at an individual level or at a sub-population level. These analyses are weakened by their cross-sectional nature. It is possible that earlier surveys may have found a relationship between migration and HIV but that the HIV positive persons then became ill and were less able to move house in the previous year. We regard this as an unlikely explanation of our findings as the two indicators of migration taken from SABSSM I were measures of movement, not just in the previous year, but over the life-course. Other population-based studies from South Africa of the individual level association between being a migrant and HIV positive, have either found a weak association [41] or no association [42].

As already noted, some initial studies that found an association between migration and HIV at the individual level made the mistake of inferring that migration was a significant determinant of HIV prevalence in populations. Predictions were made that HIV would spread rapidly in other parts of the world with high rates of human movement - such as India and China [24]. Over 250 million persons moved to a different district in China between 1979 and 2003 [28] and an estimated 307 million persons in India have moved to a different city from that where they were born [43]. Peak HIV prevalence has however remained below 0.5% in both countries [30]. In addition some authors have implied that dealing with migration was fundamental to HIV control in Southern Africa [23,44]. Calls were made to “bring the labour market closer to rural settings to arrest this phenomenon (migration’s impact on HIV spread)” [45].

## Conclusions

The results of this study do not support the notion that migration is a significant determinant of HIV prevalence when both are measured at the population level. The way migration interacts with sexual behavior to influence HIV risk in this setting is complex [41]. Although there is good evidence that migration can increase individuals’ risk of HIV in the context of specific risky practices [7,41], we could find no evidence of migration affecting HIV prevalence at a population level. The findings presented here are similar to those of studies that have found no ecological evidence to support other widely touted socioeconomic determinants of GHEs – poverty [46,47], conflict and displacement [48]. It is important to note that these findings are not at odds with the findings that migrants from high HIV prevalence regions may contribute disproportionately to the total number of HIV infections in certain low HIV prevalence regions such as Western Europe [47]. People moving from a high to a low prevalence region of a disease will increase the prevalence of that disease in the low prevalence region. It would however be inappropriate to infer that it was the population movement which led to the initial high prevalence in the high prevalence region.

Correctly identifying the main determinants of GHEs is crucial to bringing down ongoing high HIV incidence in countries such as South Africa [49-52]. The findings of this study suggest that reducing migration intensity may not be necessary to bring down HIV incidence and prevalence in countries affected by GHEs [53]. More emphasis needs to be placed on interventions and policies with solid empirical support of their efficacy in reducing HIV transmission in the setting of GHEs [54-56].

## Competing interests

The authors declare that they have no competing interests.

## Authors’ contributions

CK and ML conceived the study, and participated in its design and coordination and drafted the manuscript. RC and HV helped to draft the manuscript. CK performed the statistical analysis. All authors read and approved the final manuscript.

## Pre-publication history

The pre-publication history for this paper can be accessed here:

http://www.biomedcentral.com/1471-2334/14/350/prepub
